# Increased acetylation of microtubules rescues human tau-induced microtubule defects and neuromuscular junction abnormalities in *Drosophila*

**DOI:** 10.1242/dmm.028316

**Published:** 2017-10-01

**Authors:** Chuan-Xi Mao, Xue Wen, Shan Jin, Yong Q. Zhang

**Affiliations:** 1 Key Laboratory of Molecular and Developmental Biology, Institute of Genetics and Developmental Biology, Chinese Academy of Sciences, Beijing 100101, China; 2 College of Life Sciences, Hubei University, Wuhan, Hubei 430062, China; 3 Hubei Collaborative Innovation Center for Green Transformation of Bio-Resources, Hubei University, Wuhan, Hubei 430062, China; 4 University of the Chinese Academy of Sciences, Beijing 100049, China

**Keywords:** Acetylation, *Drosophila*, Microtubule, Tauopathy

## Abstract

Tau normally associates with and stabilizes microtubules (MTs), but is hyperphosphorylated and aggregated into neurofibrillary tangles in Alzheimer's disease and related neurodegenerative diseases, which are collectively known as tauopathies. MTs are regulated by different forms of post-translational modification, including acetylation; acetylated MTs represent a more stable microtubule population. In our previous study, we showed that inhibition of histone deacetylase 6 (HDAC6), which deacetylates tubulin at lysine 40, rescues defects in MTs and in neuromuscular junction growth caused by tau overexpression. However, HDAC6 also acts on other proteins that are involved in distinct biological processes unrelated to tubulins. In order to examine directly the role of increased tubulin acetylation against tau toxicity, we generated a site-directed *α-tubulin^K40Q^* mutation by CRISPR/Cas9 technology to mimic the acetylated MTs and found that acetylation-mimicking α-tubulin rescued tau-induced MT defects and neuromuscular junction developmental abnormalities. We also showed that late administration of ACY-1215 and tubastatin A, two potent and selective inhibitors of HDAC6, rescued the tau-induced MT defects after the abnormalities had already become apparent. Overall, our results indicate that increasing MT acetylation by either genetic manipulations or drugs might be used as potential strategies for intervention in tauopathies.

## Abstract (precis)

**Highlighted Article:** Increased acetylation of microtubules by genetic and pharmacological approaches rescues human tau overexpression-induced toxicity in *Drosophila* muscles and neurons.

## INTRODUCTION

The microtubule-associated protein tau stabilizes microtubules (MTs). However, in tauopathies, including Alzheimer's disease (AD) and frontotemporal dementia with parkinsonism linked to chromosome 17 (FTDP-17), tau is hyperphosphorylated, leading to the aggregation of tau and MT destabilization, and finally resulting in neuronal death and a reduction in brain weight and volume (Ballatore et al., 2007; Gomez-Isla et al., 1996; Huang and Mucke, 2012).

MTs are regulated by different forms of post-translational modification, including phosphorylation, glutamylation, tyrosination and acetylation. For example, acetylation occurs on lysine 40 (K40) of α-tubulin inside the MT lumen (Janke and Bulinski, 2011; Nogales et al., 1998), which is controlled by a balance of acetyltransferases and deacetylases. Two histone deacetylase-related enzymes, histone deacetylase 6 (HDAC6) and sirtuin type 2, have been found to deacetylate α-tubulin *in vivo* and *in vitro* (Hubbert et al., 2002; North et al., 2003). In the brains of AD patients, HDAC6 is significantly increased compared with the normal brain, and the tubulin acetylation is reduced in neurons carrying the tau neurofibrillary tangles (Hempen and Brion, 1996). We previously showed that ectopically expressed human tau in *Drosophila* results in decreased MT density, increased MT fragments, and more satellite boutons at neuromuscular junctions (NMJs) (Xiong et al., 2013). In *Drosophila*, HDAC6 regulates the deacetylation of α-tubulin, and we showed that null mutants of HDAC6 attenuate the tau toxicity in *Drosophila* (Xiong et al., 2013).

Specifically, loss of deacetylase activity conferred by the tubulin-specific deacetylase domain of HDAC6 is crucial for the rescue of tau-mediated MT defects (Xiong et al., 2013). HDAC6 has two deacetylase domains, DD1 and DD2. DD2 has been shown to deacetylate tubulin speciﬁcally (Haggarty et al., 2003; Kaluza et al., 2011). Introducing a mutation of H664A in HDAC6 DD2 is shown to rescue tau-mediated MT abnormalities, suggesting that amelioration of MT defects is dependent on increased MT acetylation (Xiong et al., 2013). However, HDAC6 can deacetylate multiple substrates involved in distinct biological processes unrelated to tubulins, such as tau, cortactin and the crucial chaperone, heat shock protein 90 (Cook et al., 2014; Kaluza et al., 2011; Valenzuela-Fernández et al., 2008). In addition to its deacetylase activity, HDAC6 also interacts directly with multiple proteins, including tau, the molecular chaperone p97, mDia2, ubiquitin, p150^Glued^ and protein phosphatase 1 (Boyault et al., 2006; Brush et al., 2004; Chen et al., 2005; Destaing et al., 2005; Ding et al., 2008; Seigneurin-Berny et al., 2001). Taken together, it is of importance to examine directly the role of tubulin acetylation against the toxicity of tau by mutating the K40 site of tubulin.

In this study, we aimed to determine the effect of acetylated tubulin on tauopathy. We generated site-directed *α-tubulin^K40Q^* and *α-tubulin^K40R^* mutations to mimic acetylated and non-acetylated MTs, respectively, in *Drosophila* and showed that increasing α-tubulin acetylation by either mutation or drugs can rescue tau-mediated MT defects in muscles and NMJ abnormalities in the neuronal system. Our findings suggest new targets for the development of therapeutic drugs for tauopathies.

## RESULTS

### Site-directed mutation of α-tubulin to mimic acetylated and non-acetylated MTs

There are four *α-tubulin* genes in *Drosophila*, referred to as *α1* (*CG1913*), *α2* (*CG9476*), *α3* (*CG2512*) and *α4* (*CG8308*), at chromosomal locations 84B, 85E, 84D and 67C, respectively. The genes *α1* and *α3* are constitutively expressed and differ from each other by only two amino acid substitutions (Theurkauf et al., 1986). However, *α1* is highly expressed at all developmental stages, whereas *α3* is expressed at low levels at most developmental stages (Matthews et al., 1989). By contrast, *α2* is testes specific, whereas *α4* transcripts accumulate only in ovarian nurse cells, eggs and early embryos (Theurkauf et al., 1986). Thus, αl-tubulin (hereafter referred to as α-tubulin) may be the major source for microtubules in most cells. In mammals, there are 15-20 distinct α-tubulin-encoding genes (Hall and Cowan, 1985). Thus, owing to functional redundancy, it is difficult to determine the effects of acetylated MT on physiological development and pathology *in vivo.*

**Fig. 1.**
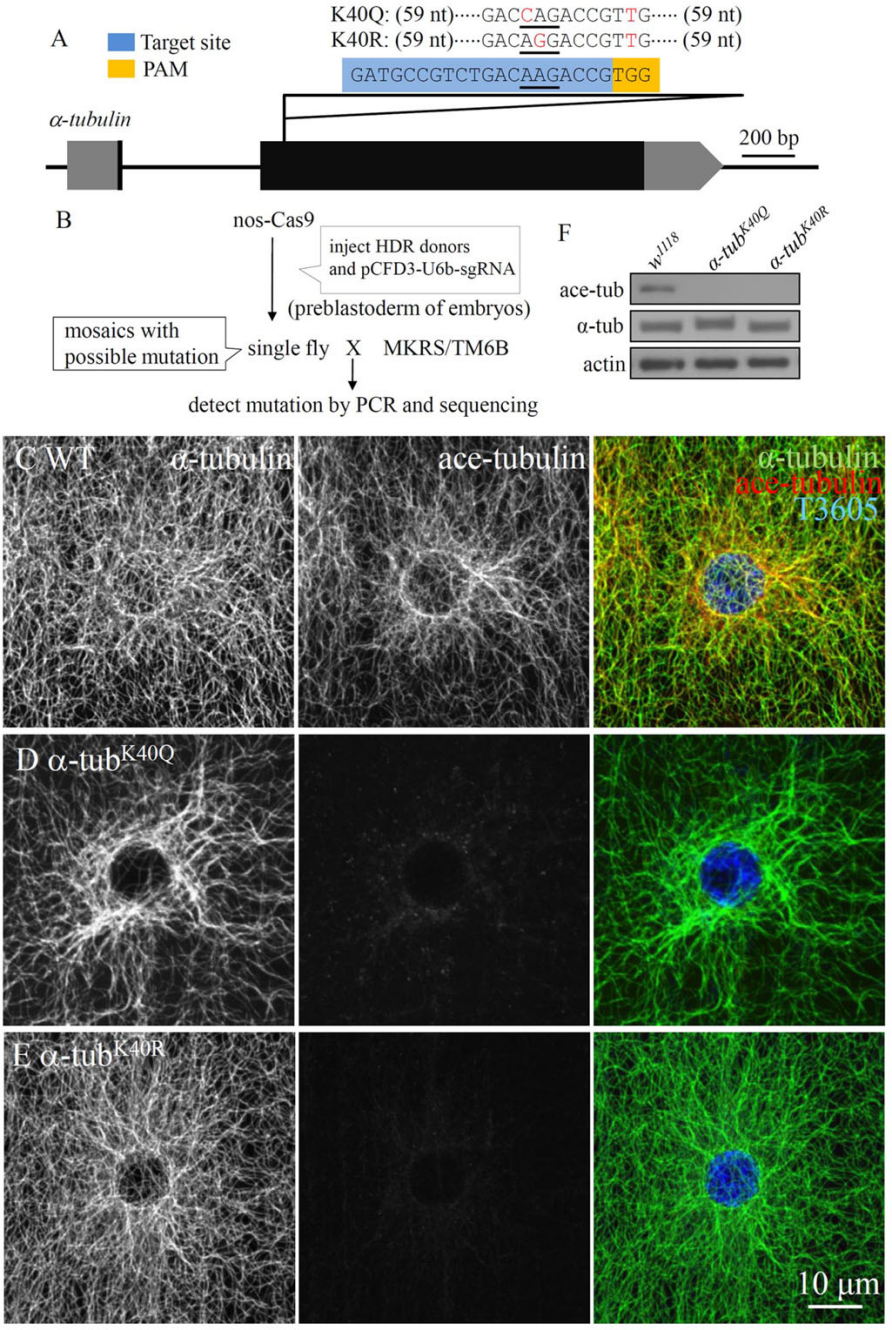
**CRISPR/Cas9-mediated mutagenesis of *α-tubulin*.** (A) The intron-exon organization of *α-tubulin* (*CG1913*). Exons and untranslated region (UTR) are shown as black and grey blocks, respectively. The sgRNA target site is in blue. PAM, protospacer-adjacent motif highlighted in yellow. For targeted point mutation, the lysine (K) 40 codon, AAG, was replaced with the glutamine (Q) codon, CAG, or arginine (R) codon, AGG. (B) Cross scheme and screening procedures. HDR, homology-directed repair. (C-E) The larval muscles co-stained with antibodies against total tubulin, acetylated tubulin and T3605, which labels the nucleus in wild type (C), *α-tub^K40Q^* (D) and *α-tub^K40R^* (E). Scale bar: 10 μm. (F) Western blot analysis of acetylated and total α-tubulin in larval muscles of wild type, *α-tub^K40Q^* and *α-tub^K40R^* mutants. Actin was used as a loading control.

To define the role of acetylated tubulin on MT dynamics better, we set out to make point mutations of the widely and abundantly expressed α-tubulin. Glutamine is hydrophilic and uncharged, and similar to acetylated lysine, whereas arginine has a positively charged side chain like lysine but cannot be acetylated (Kim et al., 2006; Li et al., 2012). Using CRISPR/Cas9-mediated targeted mutagenesis, we generated two different *α-tubulin* mutations, in which α-tubulin K40 was substituted with glutamine (K40Q) or arginine (K40R) to mimic acetylated and non-acetylated tubulin, respectively (Fig. 1A,B). Both mutants showed normal development and fertility in both sexes. Staining with antibodies against total α-tubulin in *α-tub^K40Q^* mutants revealed that there were more MT bundles in the perinuclear area of muscle cells compared with wild type (Fig. 1C,D). However, we did not find an apparent difference in MT network in muscle cells between *α-tub^K40R^* and wild type (Fig. 1C,E). In both *α-tub^K40Q^* and *α-tub^K40R^* mutants, very weak or no staining for acetylated α-tubulin was detected in muscles (Fig. 1D,E) or within the neuronal system (data not shown). The results of western blotting did not reveal any signals for expected acetylated α-tubulin in *α-tub^K40Q^* or *α-tub^K40R^* mutant muscles (Fig. 1F). Notably, the α-tubulin band of *α-tub^K40Q^* was slightly higher than the wild type. These results demonstrate that the antibody against acetylated tubulin did not recognize either *α-tub^K40Q^* or *α-tub^K40R^* mutants, and that α-tubulin encoded by *CG1913* remained the major, if not the only tubulin within the muscles.

### MTs carrying *α-tub^K40Q^* mutation are more resistant to cold treatment

Increased acetylation has been associated with MT stabilization (Janke and Bulinski, 2011). However, it was found by kinetic analysis of polymerization and depolymerization that isolated acetylated and non-acetylated MTs have similar stabilities *in vitro* (Maruta et al., 1986). Thus, the effects of acetylation on MT dynamics remain uncertain.

**Fig. 2.**
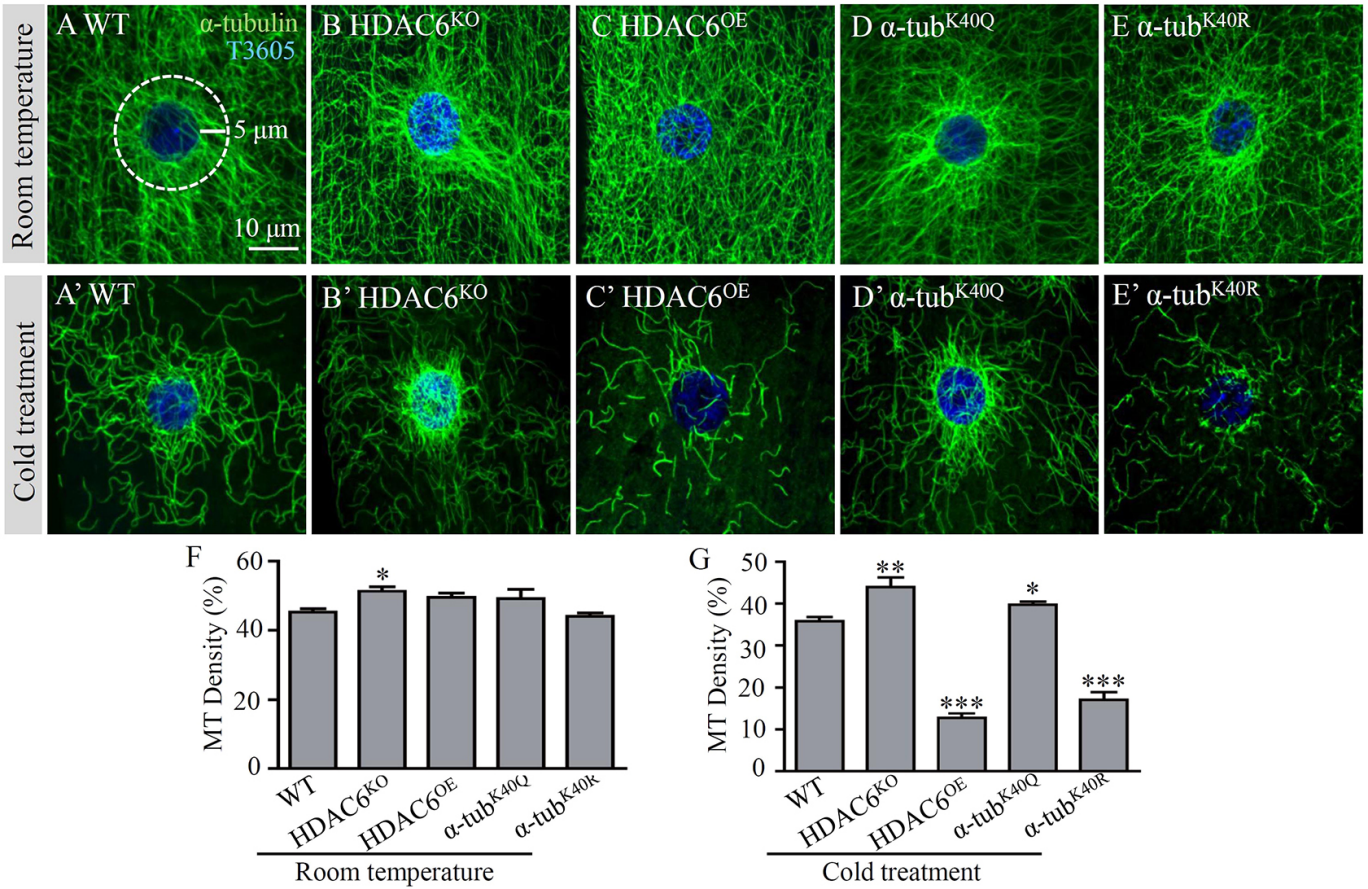
**Acetylated MTs are more resistant to cold treatment.** Third instar larval muscles were co-stained with antibodies against total tubulin (green) and a nuclear dye T3605 (blue). (A,B,C,D,E) Muscle cells of wild type (A), *HDAC6^KO^* null mutants (B), HDAC6-overexpressing animals (C), *α-tub^K40Q^* (D) and *α-tub^K40R^* (E) mutant larvae maintained at 25°C. (A′,B′,C′,D′,E′) Muscle cells from larvae of the different genotypes treated on ice for 30 min to depolymerize MTs and stained with anti-α-tubulin (green) and T3605 (blue). Scale bar: 10 μm. (F,G) Quantification of perinuclear MT densities in muscle cells of different genotypes treated at room temperature (F) or on ice for 30 min (G) before dissection. *n*=12 muscle cells for each genotype. *P<0.05, **P<0.01, ***P<0.001 (one-way ANOVA). Error bars indicate s.e.m.

Cold treatment is widely used to induce MT depolymerization in live cells (Chu et al., 2011; Tala et al., 2014). To determine whether the *α-tub^K40Q^* mutation increased the stability of MTs, we treated live larvae on ice for 30 min before dissection and immunostaining. At room temperature, the MT network appeared largely normal in different genotypes except that there were thick MT bundles in *HDAC6* null (*HDAC6^KO^*) mutant cells, in which the perinuclear MT density was mildly but significantly increased compared with wild type (Fig. 2A–F). Upon cold treatment, the MT network in muscle cells of wild type was greatly reduced, and only a residual MT network could be observed in muscles (Fig. 2A,A′). Compared with wild type, *HDAC6^KO^* and *α-tub^K40Q^* mutants exhibited significantly denser perinuclear MTs after cold treatment (∼23% increase, *P*<0.01 for *HDAC6^KO^* and 11% increase, *P*<0.05 for *α-tub^K40Q^*; Fig. 2A′,B′,D′,G). On the contrary, in HDAC6-overexpressing larvae and *α-tub^K40R^* mutants, the density of the MT network was significantly reduced compared with the wild type after cold treatment (36 and 48% of the wild-type density, respectively; *P*<0.001 for both), and the MT fibres were much shorter and sparser (Fig. 2A′,C′,E′,G). In summary, the MTs in *HDAC6^KO^* and *α-tub^K40Q^* mutants, which have a higher level of acetylation and acetylation-mimicking MTs, respectively, are more resistant to cold-induced MT depolymerization, whereas the MTs in *HDAC6^OE^* and *α-tub^K40R^* mutants, with un-acetylated α-tubulin, are more labile on cold treatment.

### *α-tub^K40Q^* mutation rescues the MT defects and NMJ growth anomalies caused by *tau^V337M^* overexpression

We previously reported that HDAC6 mutations and inhibitors increase α-tubulin acetylation and rescue the tau toxicity induced by overexpression of *tau^V337M^* (Xiong et al., 2013), demonstrating the importance of α-tubulin acetylation in inhibiting tau toxicity. We previously showed that *HDAC6* mutations do not affect tau phosphorylation in *Drosophila* (Xiong et al., 2013), although HDAC6 inhibition reduces tau hyperphosphorylation in mice (Zhang et al., 2014). Given that HDAC6 has multiple substrates and interacting proteins (Cook et al., 2014; Ding et al., 2008; Kaluza et al., 2011; Valenzuela-Fernández et al., 2008), it remains possible that mechanisms other than tubulin acetylation might contribute to the rescue effect.

**Fig. 3.**
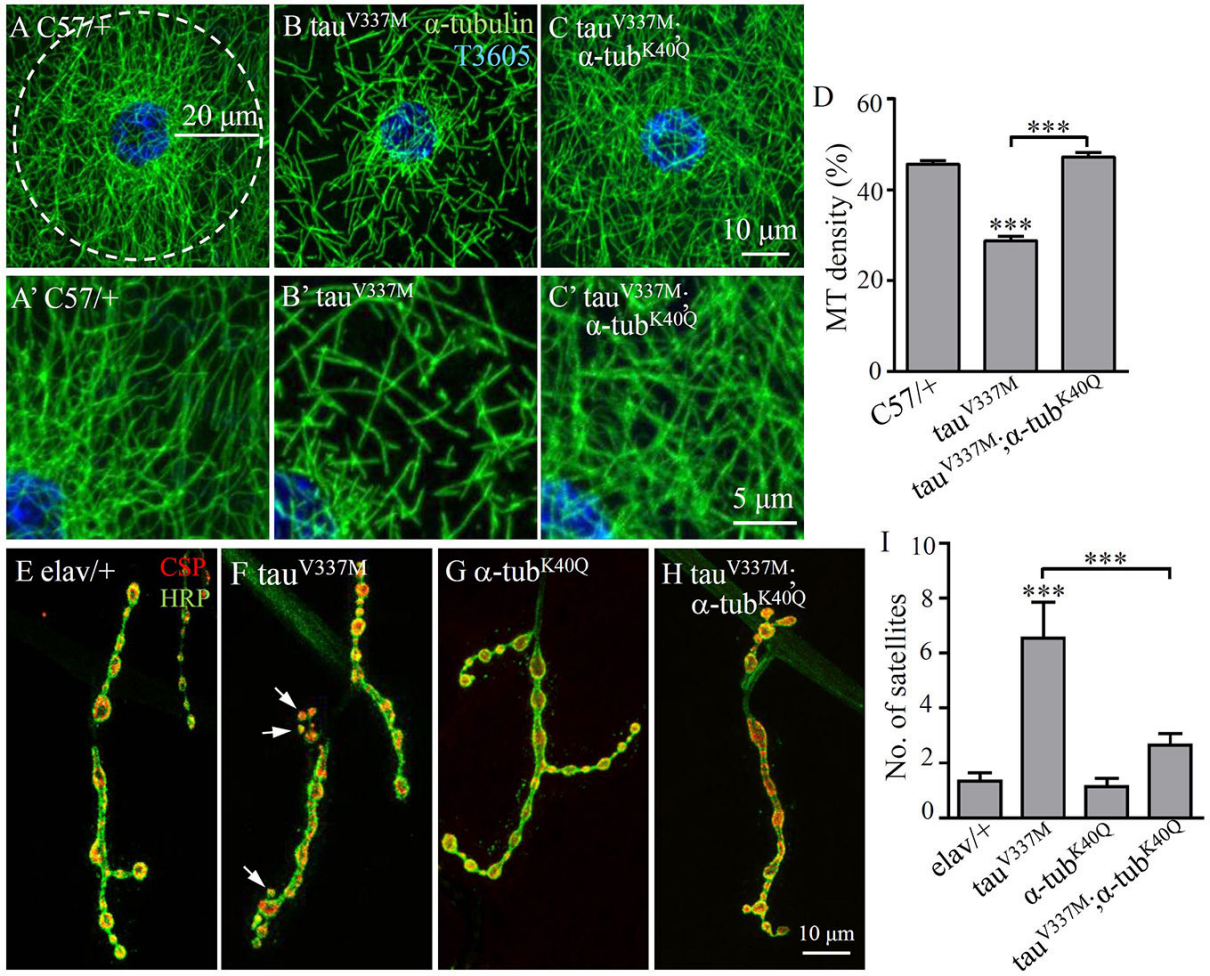
***α-tub^K40Q^* antagonizes tau overexpression in regulating MT network formation in muscle cells and NMJ growth.** (A-C′) Third instar larval muscles double-labelled with anti-tubulin (green) to reveal MT network and nuclear T3605 (blue). A′-C′ are magnified images of the top-right of the corresponding A-C. (A,A′) Genetic control *C57-Gal4*/*+*. (B,B′) Muscular overexpression of *tau^V337M^* (*C57-Gal4/tau^V337M^*) resulted in decreased MT density. (C,C′) Overexpression of *tau^V337M^* in *α-tub^K40Q^* background (*C57-Gal4/tau^V337M^; α-tub^K40Q^*). *α-tub^K40Q^* mutation rescued MT defects caused by *tau^V337M^* overexpression. (D) Quantification of MT densities in different genotypes. *n*=15 cells for each genotype. (E-H) Representative muscle four NMJs from wandering third instar larvae double-labelled with anti-CSP (red) and anti-HRP (green) to reveal synaptic vesicles and the neuronal membrane, respectively. (E) Genetic control *elav-Gal4*/*+*. (F) Neuronal overexpression of *tau^V337M^* (*elav-Gal4/tau^V337M^*). (G) *α-tub^K40Q^*. (H) Overexpression of *tau^V337M^* in *α-tub^K40Q^* background (*elav-Gal4/tau^V337M^; α-tub^K40Q^*). The *α-tub^K40Q^* mutation rescued the increased number of satellite boutons caused by neuronal overexpression of *tau^V337M^*. Arrows in F indicate satellite boutons. Scale bars: 20 μm in A; 10 μm in C and H; 5 μm in C′. (I) Quantification of the number of satellite boutons in different genotypes. *n*=12 NMJs for each genotype. ***P<0.001 (one-way ANOVA). Error bars indicate s.e.m.

To define the effects of MT acetylation on MT defects caused by tau overexpression, we expressed human FTDP-17-associated V337M mutant tau in *Drosophila* and analysed genetic interactions between *α-tub^K40Q^* and the ectopically expressed tau by quantifying the density of perinuclear MT in larval muscle cells. Muscle-specific overexpression of *tau^V337M^* (*C57-Gal4*/+, *tau^V337M^*) led to significantly reduced perinuclear MT density, with sparser and shorter fibres compared with the genetic control (*C57-Gal4*/+; Fig. 3A,A′,B,B′). Specifically, the percentage of the tubulin-positive area in the defined perinuclear area was 29% in muscle cells overexpressing *tau^V337M^* (Fig. 3B,B′,D), compared with 46% in the genetic control (*P*<0.001; Fig. 3A,A′,D). The decrease of perinuclear MT density caused by *tau^V337M^* overexpression was fully rescued by the *α-tub^K40Q^* mutation (47%; *P*<0.001; Fig. 3C,C′,D), demonstrating that the *tau^V337M^*-induced MT defects could be fully rescued by acetylation-mimicking α-tubulin.

In order to investigate the effect of acetylated microtubules on tau toxicity in other cell types, we ectopically expressed *tau^V337M^* in neurons driven by the pan-neuronal *elav-Gal4*. Our results showed that neuronal overexpression of *tau^V337M^* led to more satellite boutons at the NMJ (6.55±1.32) compared with the genetic control (1.33±0.31; *P*<0.001; Fig. 3E,F,I), consistent with previous reports (Chee et al., 2005; Xiong et al., 2013). The excess satellite boutons caused by neuronal overexpression of *tau^V337M^* were rescued by the homozygous *α-tub^K40Q^* mutation (2.64±0.43; *P*<0.001; Fig. 3F,H,I), which alone showed normal NMJs (1.14±0.29; Fig. 3G,I). Together, these results show that acetylation-mimicking α-tubulin mutation rescues tau-induced toxicity in both neuronal and non-neuronal cells.

We also performed a genetic interaction assay between *tau^V337M^* and *α-tub^K40R^*. To our surprise, *α-tub^K40R^* partly rescued MT defects, and completely reversed more satellite boutons induced by tau overexpression (Fig. S3). Increased MT acetylation and *α-tub^K40Q^* mutation might rescue tau-induced defects, probably by promoting motor-based transport (Reed et al., 2006; Song and Brady, 2015), whereas *α-tub^K40R^* rescued tau-induced defects by a presently unknown mechanism. We note that α-tub^K40R^ has a positively charged guanidino group, which might affect the *in vivo* function of microtubules, leading to the rescue of tau-induced MT defects. In summary, the mimic of non-acetylated tubulin, α-tub^K40R^, rendered MT more labile on cold treatment (Fig. 2E′), but it did not perform as non-acetylated tubulins in enhancing tau toxicity in muscles (Fig. S3G).

### Pharmacological inhibition of the tubulin-specific deacetylase activity of HDAC6 rescues MT defects caused by *tau^V337M^* overexpression

Mounting evidence supports the notion that administration of HDAC6 inhibitors and MT-stabilizing drugs improve transport defects and cognition in transgenic mouse models of tauopathy (Zhang et al., 2005, 2014). In *Drosophila*, the tau-induced MT defects are significantly rescued by tubacin, an inhibitor of the tubulin-specific deacetylase activity of HDAC6 (Xiong et al., 2013). Although tubacin was previously added to medium where eggs were laid and larvae developed (Xiong et al., 2013), it was not known whether the drug protects MT integrity, reverses MT defects, or a combination of both. To examine the possibility of a potential use in medicine, we set out to determine whether the tau toxicity could be rescued by HDAC6 inhibitors after the MT defects had already formed.

**Fig. 4.**
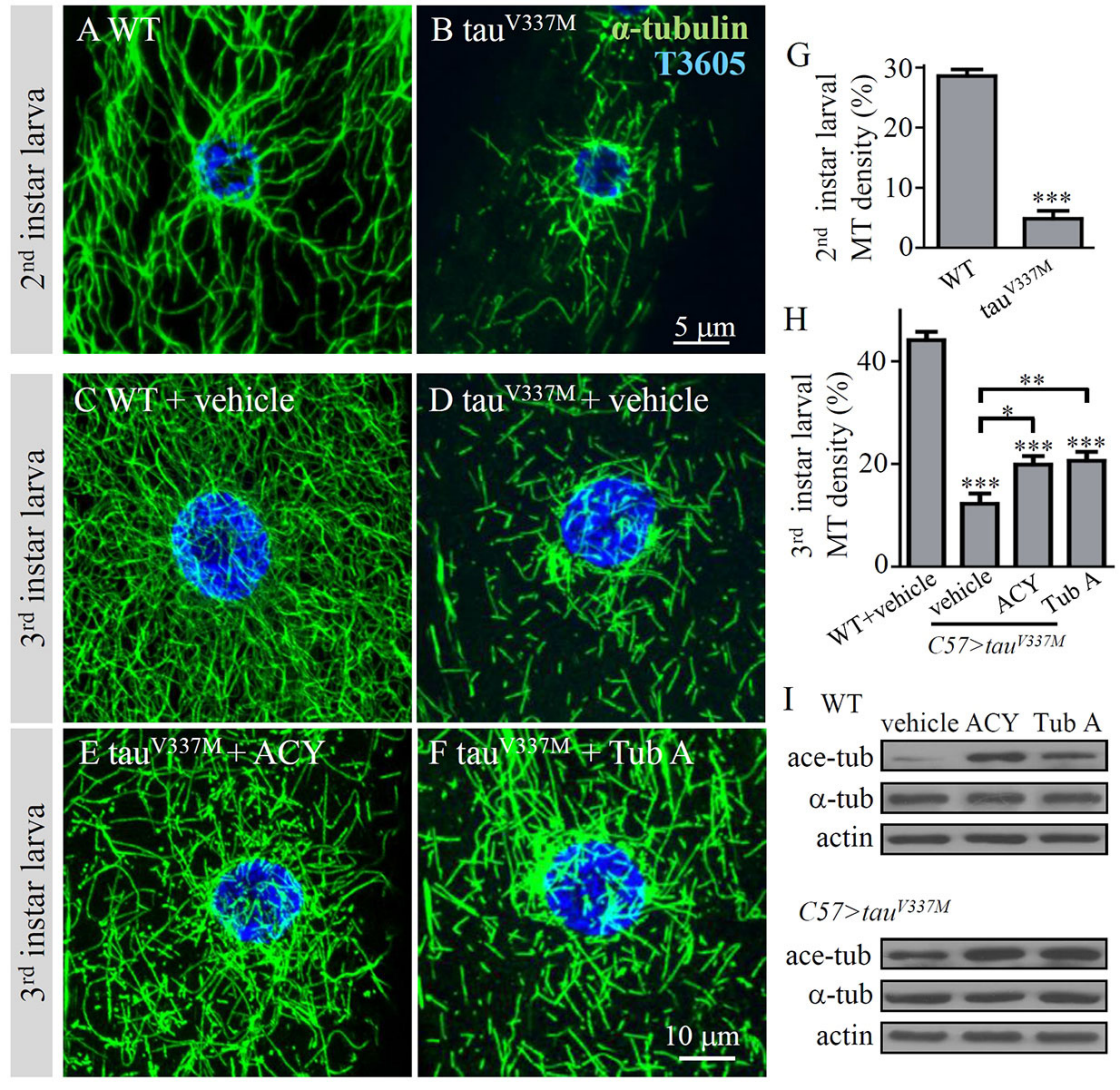
**Inhibition of HDAC6 by ACY-1215 or tubastatin A increases acetylation of tubulin and rescues MT defects caused by tau overexpression.** (A-F) *Drosophila* second or third instar larval muscles co-stained with anti-α-tubulin (green) to show MT network and T3605 (blue) to show the nucleus. (A) The MT network at second instar larval stage. (B) The MT network was disrupted at second instar larval stage in *tau^V337M^*-overexpressing larvae. Scale bar: 5 μm. (C-F) The third instar larval of different genotypes were fed cornmeal medium containing vehicle DMSO (C,D), ACY-1215 (E) or tubastatin A (F) from second instar larval stage. Scale bar: 10 μm. (G) Quantification of MT densities in the perinuclear area of second instar larvae in wild-type and *tau^V337M^*-overexpressing larvae. *n*=8 cells for each genotype. ***P<0.001 (*t*-test). Error bars indicate s.e.m. (H) Quantification of MT densities in the perinuclear area of third instar larval muscles from different genotypes treated with or without drugs. Comparisons were made between each genotype and the wild-type controls, as well as between specific genotypes (bracketed). *n*=16 cells for each genotype. *P<0.05, **P<0.01, ***P<0.001 (one-way ANOVA). Error bars indicate s.e.m. (I) ACY-1215 or tubastatin A treatment did not alter the level of total α-tubulin but increased the level of acetylated-tubulin in both wild-type and tau-overexpressing muscles.

The use of tubacin in disease models has helped to validate HDAC6 as a drug target, but its non-drug-like structure, high lipophilicity and tedious synthesis together make it more useful as a research tool than a drug (Butler et al., 2010; Haggarty et al., 2003). ACY-1215 and tubastatin A are newly developed potent and highly selective inhibitors of the tubulin-specific deacetylase activity of HDAC6 and are more readily accessible than tubacin (Butler et al., 2010; Santo et al., 2012). We examined whether ACY-1215 and tubastatin A could rescue the already formed MT defects caused by *tau^V337M^* overexpression. Overexpression of *tau^V337M^* by *C57-Gal4* led to apparent MT defects in the second instar larvae (Fig. 4A,B,G). As expected, treatment with 100 μM ACY-1215 or tubastatin A significantly increased the levels of acetylated α-tubulin in wild-type and *tau^V337M^* overexpression animals (Fig. 4I). Feeding larvae with 100 μM ACY-1215 and tubastatin A from the second larval stage mildly but significantly rescued the MT defects (*P*<0.05 and *P*<0.01, respectively, compared with the vehicle-fed larvae; Fig. 4C–F,H). As a control, the drug treatment of wild-type larvae did not affect the MT network or density (Fig. S1). In addition, a lower dose of 50 μM tubastatin A led to no obvious rescue, suggesting that the rescue effect of MT defects caused by *tau^V337M^* overexpression was dose dependent (Fig. S2). Together, our results suggest that ACY-1215 and tubastatin A might be therapeutically beneficial in tauopathies.

### ACY-1215 and tubastatin A rescue NMJ abnormality caused by *tau^V337M^* overexpression

**Fig. 5.**
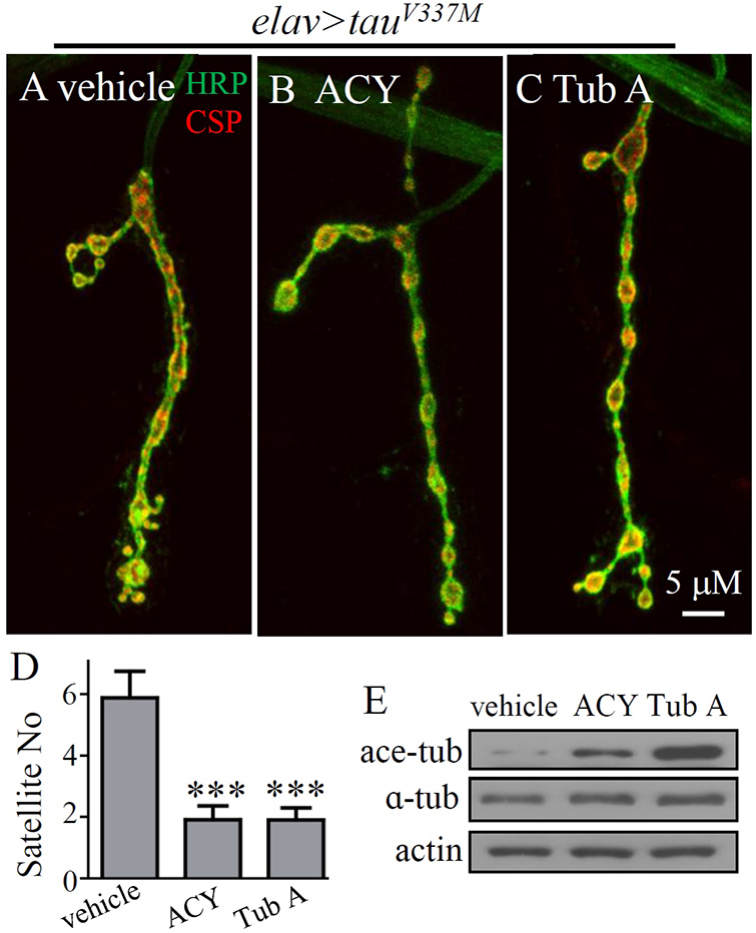
**Inhibition of HDAC6 by ACY-1215 and tubastatin A rescues the increased number of satellite boutons induced by neuronal overexpression of tau.** (A-C) Larvae overexpressing *tau^V337M^* were fed cornmeal medium containing vehicle (DMSO), ACY-1215 or tubastatin A (100 μM) from second instar larval stage and examined at late third instar larval stage. The increased satellite boutons caused by *tau^V337M^* overexpression were partly reversed by both ACY-1215 (B) and tubastatin A (C). Scale bar: 5 μm. (D) Quantification of the number of satellite boutons in *tau^V337M^*-overexpressing larvae with or without drug treatment. *n*=16 NMJs for each genotype. ***P<0.001 (one-way ANOVA). Error bars indicate s.e.m. (E) In larval brains, treatment with ACY-1215 or tubastatin A did not alter the level of total α-tubulin but increased the level of acetylated tubulin.

As described above, ACY-1215 and tubastatin A rescued the already formed MT defects in muscles caused by muscle-specific *tau^V337M^* overexpression (Fig. 4). We next examined the rescue effects of the two drugs in the neuronal system. We fed larvae overexpressing *tau^V337M^* in neurons with ACY-1215 and tubastatin A from the second instar larval stage, and checked the phenotype of the NMJ at the third instar larval stage. Although no obvious defects were observed in second instar larvae expressing *tau^V337M^* by *elav-Gal4* (data not shown), there were significantly more satellite boutons at the NMJs of third instar larvae overexpressing *tau^V337M^* pan-neuronally compared with the genetic controls (Fig. 3E,F,I). The anomaly of excess satellite boutons attributable to neuronal overexpression of *tau^V337M^* was fully reversed by ACY-1215 (1.91±0.45; *P*<0.001) and tubastatin A (1.91±0.39; *P*<0.001) compared with the vehicle-treated mutants (Fig. 5A–D). These results show that inhibition of HDAC6 and increased acetylation of MTs by ACY-1215 or tubastatin A rescue NMJ growth defects by inhibiting the toxicity caused by neuronal overexpression of *tau^V337M^*.

## DISCUSSION

### Increased acetylation of α-­tubulin K40 promotes MT stability

Acetylation of α-­tubulin K40 on the luminal face of MTs is an important post-translational modification of tubulin and is common on stable MTs in most cell types, yet the physiological role of α-­tubulin K40 acetylation remains elusive. Besides α-­tubulin K40, there are several additional lysine residues (K394, K60 and K370 of α-tubulin and K58, K103, K154 and K252 of β-tubulin) reported to be putative sites of acetylation (Liu et al., 2015). However, the functions of these seven sites have not been identified. Acetylation of α-tubulin K40 is not required for survival in protists, such as *Tetrahymena* and *Chlamydomonas*, but it is important in various biological processes (Akella et al., 2010; Gaertig et al., 1995; Kozminski et al., 1993). For example, MTs with reduced acetylation or carrying a K40R substitution in α-tubulin of *Tetrahymena* are more dynamic (Akella et al., 2010). In cultured fibroblasts, acetylation of α-tubulin K40 is required for contact inhibition of proliferation and cell-substrate adhesion (Aguilar et al., 2014). Neurons overexpressing a K40A mutant, which mimic non-acetylated α-tubulin, show altered motor-based trafficking and cell differentiation in mice (Creppe et al., 2009).

Our mutational analysis showed that acetylation of α-­tubulin is not essential for survival in *Drosophila*. There were no obvious differences in lifespan, locomotion or fertility between wild type and *α-­tub^K40Q^* or *α-­tub^K40R^* mutants. However, there were increased MT bundles in the perinuclear area in *α-­tub^K40Q^* muscle cells (Fig. 1D), and *α-­tub^K40Q^* MTs were more resistant to cold-induced disassembly (Fig. 2D′,G). On the contrary, MT fibres were much shorter and sparser in *α-­tub^K40R^* mutants upon cold treatment (Fig. 2E′,G), consistent with a previous report (Akella et al., 2010), although there was no significant difference in MT network between *α­-tub^K40R^* mutants and wild type at room temperature (Fig. 1E; Fig. 2E,F). Thus, our findings support the hypothesis that acetylation of α-­tubulin K40 promotes MT bundle formation and enhances MT stability upon cold treatment.

### *α-tub^K40Q^* mutation rescues MT defects caused by *tau^V337M^* overexpression

MT defects, a hallmark of tauopathies, contribute directly to neurodegeneration (Ballatore et al., 2007). Previous studies in both cell cultures and primary culture neurons reveal that overexpressed tau shows reduced MT binding to motor proteins and inhibits transport of cellular components, which lead to MT disruption and synaptic decay (Mandelkow et al., 2004; Thies and Mandelkow, 2007). However, how the overexpressed tau leads to MT defects *in vivo* remains poorly understood. Our previous work showed that increased MT acetylation in *HDAC6* null mutants rescued tau-induced MT defects in both muscles and neurons (Xiong et al., 2013). In mammals, HDAC6 binds with tau directly, maintains site-specific tau phosphorylation and promotes tau accumulation (Cook et al., 2012; Ding et al., 2008). Thus, it is possible that *HDAC6* mutations rescue tau-mediated MT defects by promoting degradation of phosphorylated tau. However, *HDAC6* null mutation does not reduce the level of phosphorylated tau in *Drosophila* (Xiong et al., 2013).

Our previous study supports the hypothesis that increased MT acetylation by inhibition of HDAC6 activity rescues tau-induced defects (Xiong et al., 2013), but other functions of HDAC6 might also have contributed to this rescue ability. Here we show, for the first time, that the acetylation-mimicking *α-tub^K40Q^* mutation rescues tau-induced pathologies (Fig. 3). How do the acetylation-mimicking *α-tub^K40Q^* mutation and increased MT acetylation rescue tau-induced MT defects? Neurons with long axons are likely to be highly sensitive to defects in motor proteins and their microtubule tracks. Trafficking defects have been implicated in various neurodegenerative diseases, including AD, amyotrophic lateral sclerosis and lissencephaly (Chevalier-Larsen and Holzbaur, 2006; Duncan and Goldstein, 2006; Keays et al., 2007). In cultured cells, tau overexpression results in blockage of transport, most probably as a result of excessive binding of tau to MTs, which not only prevents the transport of vesicles, but also hinders the binding of motor proteins to MTs (Ballatore et al., 2007; Dixit et al., 2008; Thies and Mandelkow, 2007). MT acetylation was reported to enhance kinesin-based transport, as inhibition of tubulin deacetylase modestly enhances kinesin binding to MTs and redistributes a putative kinesin cargo (Reed et al., 2006; Song and Brady, 2015). However, acetylation and kinesin motility and accumulation are not shown to correlate *in vivo* (Hammond et al., 2010), and tubulin acetylation alone does not affect kinesin-1 velocity and run length *in vitro* (Walter et al., 2012). Thus, whether increased MT acetylation rescues tau-induced MT defects by regulating motor-based transport remains to be investigated further.

Alternatively, MT acetylation might affect protein interactions with MTs, as acetylated MTs are favoured for severing by katanin (Mao et al., 2014; Sudo and Baas, 2010). By contrast, axonal MTs are less sensitive to katanin, as a result of the fact that axonal tau protects acetylated MTs from katanin (Yu et al., 2008). Thus, the acetylation level and the extent to which MTs are bound by MT-associated proteins determine the stability and dynamics of MTs in physiological and pathological conditions. It will be interesting to test whether katanin plays a role in the MT acetylation-mediated rescue of tau toxicity in both neuronal and non-neuronal systems.

## MATERIALS AND METHODS

### *Drosophila* stocks and husbandry

Flies were cultured on standard cornmeal medium at 25°C unless otherwise specified. *w^1118^* was used as the wild-type control. Other stocks used included the muscle-specific *C57*-*Gal4* (from V. Budnik, Univeristy of Massachusetts, Worcester, MA, USA), pan-neuronal *elav*-*Gal4* and *nos-Cas9* (both from Bloomington Stock Center). *UAS*–*tau^V337M^* was from M. Feany (Harvard Medical School, Boston, MA, USA). *HDAC6* null mutant (*HDAC6^KO^*) and *UAS-HDAC6* were from R. Jiao (Institute of Biophysics, CAS, Beijing, China; Du et al., 2010).

### Site-directed mutation of the *α-tubulin* locus

We generated two site-directed mutations into *α-tubulin* (*CG1393*) from wild-type K40 to glutamine (Q) and arginine (R) to mimic the acetylated and non-acetylated protein, respectively (Kim et al., 2006; Li et al., 2012). The CRISPR/Cas9-mediated targeted mutagenesis of *α-tubulin* was performed largely according to previously published homology-directed repair procedures (Port et al., 2014). Efficient target recognition by the CRISPR/Cas9 system requires 20 nucleotides (nt) of homology between the single-guide RNA (sgRNA) and its genomic target (Gratz et al., 2013). Cleavage also requires that the 3′ end of the genomic target sequence contains a 3 bp proto-spacer adjacent motif (PAM) sequence, TGG, which differentiates self from invading DNA (Fig. 1A) (Jinek et al., 2012). Generation of *α-tubulin* sgRNA was performed by cloning sgRNA (a single synthetic guide RNA, gatgccgtctgacaagaccg) into the pCFD3-dU6 vector (49410; Addgene). This designed sgRNA recognized the *α-tubulin* DNA sequence at the target site nearby to the codon of α-tubulin K40 (Fig. 1A). The 131 bp single-stranded DNA oligonucleotide donor (ssODN) containing the site-directed mutation was synthesized. The ssODN sequence, cctgctgggagctctactgcttggagcacggcatccagcccgatggccagatgccgtctgaccag (or agg) accgttggcggaggtgatgactcgttcaacaccttcttcagcgagactggagctggcaagcacgtg (131 bp, single-stranded DNA), harbours a site-directed mutation within the sgRNA-*α-tubulin* target site, and a same-sense mutation in the PAM sequence (Fig. 1A). The donor DNA with the site-directed mutation (1 µg/µl) and the pCFD3-U6b-sgRNA-*α-tubulin* (400 ng/µl) were injected into the *nos-Cas9* transgenic line (Fig. 1B), and the eclosed adults were crossed with *MKRS/TM6B*. The *α-tubulin* in the offspring was sequenced to identify whether the flies carried a mutation or not (Fig. 1B).

### Immunochemical analyses and confocal microscopy

Western analysis was performed as described previously (Jin et al., 2009; Mao et al., 2014). Third instar larval carcasses, larval brains and ventral nerve cords were dissected in PBS, followed by homogenization in a lysis buffer [50 mM Tris-HCl, (pH 7.4), 150 mM NaCl, 1% NP-40 and 0.1% SDS]. Blots were first probed with primary antibodies anti-α-tubulin (1:10,000; mAb B-5-1-2; Sigma-Aldrich), anti-acetylated tubulin (1:10,000; mAb 6-11B-1; Sigma-Aldrich) and anti-actin (1:50,000; mAb 1501; Chemicon), followed by incubation with horseradish peroxidase (HRP)-coupled secondary antibodies. Protein bands were visualized by a chemiluminescence method (ECL Kit; Amersham).

For immunostaining of muscles and NMJs, we used the following primary antibodies: anti-α-tubulin (1:800; mAb B-5-1-2; Sigma-Aldrich), anti-acetylated tubulin (1:800; mAb 6-11B-1; Sigma-Aldrich), anti-cysteine string protein [CSP; 1:200; 6D6; Developmental Studies Hybridoma Bank (DSHB)], and FITC-conjugated goat anti-HRP (1:100; Jackson ImmunoResearch). To examine the MT network in muscles, muscle 2 in abdominal segment A4 was analysed, as it has fewer tracheal branches to obscure the viewing of MTs (Mao et al., 2014). Nuclei were visualized by TO-PRO(R) 3 iodide (T3605; 1:1000; Invitrogen) staining for 1 h at room temperature. Images were collected with an Olympus FV10-ASW confocal microscope and analysed using projections from complete *z*-stacks through the entire muscle four NMJ of the abdominal segment A3. Synaptic boutons were defined according to co-staining signals by anti-HRP, which labels neuronal membranes, and anti-CSP, which detects presynaptic vesicles. Satellite boutons were defined as boutons of smaller size than the adjacent mature boutons along NMJ branches.

### Quantification of perinuclear MT density

Quantification of MT density in muscles was performed as previously described (Jin et al., 2009; Mao et al., 2014; Xiong et al., 2013). All images were projections of serial stacks through the muscle cell. The perinuclear areas were defined as the coverage that spans 5 or 20 μm around T3605-labelled nuclei. Tubulin staining signals within the perinuclear area from muscle 2 of abdominal segment A4 were calculated using ImageJ 3.0. The software reports the ratio of the tubulin-positive area divided by the total perinuclear area.

### MT cold treatment assay

Live third instar larvae were put on ice for 30 min to depolymerize MTs. After dissection in cold Ca^2+^-free HL3.1 saline (70 mM NaCl, 5 mM KCl, 20 mM MgCl_2_, 10 mM NaHCO_3_, 115 mM sucrose, 5 mM trehalose and 5 mM HEPES) and fixation by paraformaldehyde, immunofluorescence staining was performed as previously described (Jin et al., 2009; Mao et al., 2014).

### Pharmacological treatment of larvae with ACY-1215 and tubastatin A

Viable second instar larvae (48-72 h after egg laying) of *elav*-*Gal4*>*UAS-tau^V337M^* or control were removed from normal medium to the pre-prepared medium containing drug or DMSO vehicle control. ACY-1215 and tubastatin A (Selleck Company) were prepared as 10 mM stocks in DMSO and then added at a final concentration of 100 μM. Wandering late third instar larvae were dissected for immunostaining or western analysis.

### Statistical analyses

All statistical comparisons were performed using GraphPad InStat 5 software. *P-*values were calculated by one-way ANOVA. Comparisons were made between a specific genotype and the wild-type control (asterisks are located above a column) or between two specific genotypes (asterisks are located above a bracket).

## Supplementary Material

Supplementary information
