# Supplementary material for: Increased acetylation of microtubules rescues human tau-induced microtubule defects and neuromuscular junction abnormalities in Drosophila
Source: Dis Model Mech. 2017 Oct 1;10(10):1245–52. doi: 10.1242/dmm.028316 (PMC5665452; doi:10.1242/dmm.028316)
Supplement: Supplementary information [file dmm-10-028316-s1.pdf]

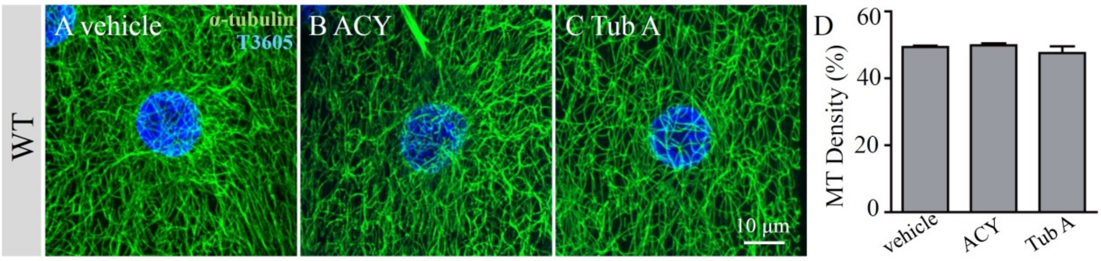

Fig. S1. Inhibition of HDAC6 by ACY-1215 or tubastatin A does not affect MT network or density. (A-C) Wild-type 3rd instar larval muscles co-stained with anti-α-tubulin (green) to show MT network and T3605 (blue) to show the nucleus. The 2nd instar larvae were fed cornmeal medium containing vehicle DMSO (A), 100 μM ACY-1215 (B) or 100 μM tubastatin A (C) until analysis at the 3rd instar larval stage. (D) Quantification of MT densities in 20 μm perinuclear area of 3rd instar larvae in wild type.

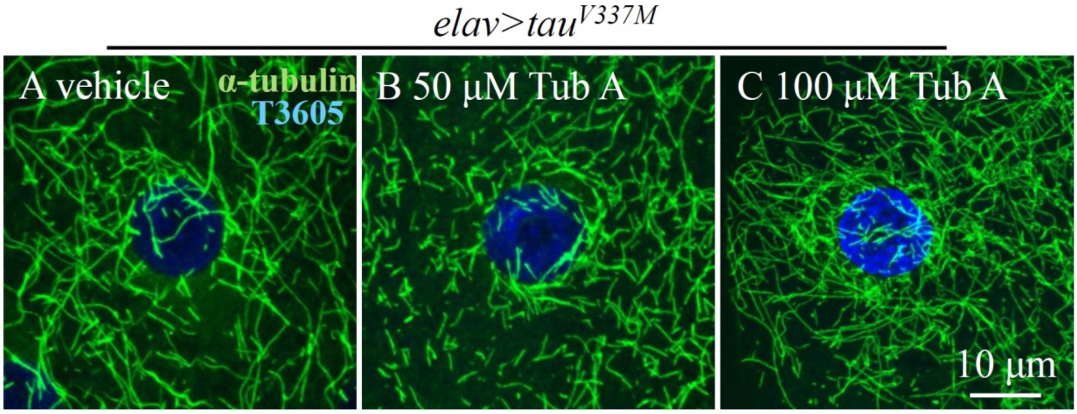

Fig. S2. Inhibition of HDAC6 rescues MT defects caused by tau^{V337M} overexpression in a dose-dependent manner. (A-C) Third instar larval muscles double-labeled with anti-tubulin to reveal MT network (green) and T3605 to reveal nucleus (blue). The larvae overexpressing tau^{V337M} were fed cornmeal medium containing vehicle DMSO (A), 50 μM tubastatin A (B) or 100 μM tubastatin A (C) from 2nd instar larval stage. Higher dose of tubastatin A at 100 μM showed a better rescue of MT defects than that at 50 μM.

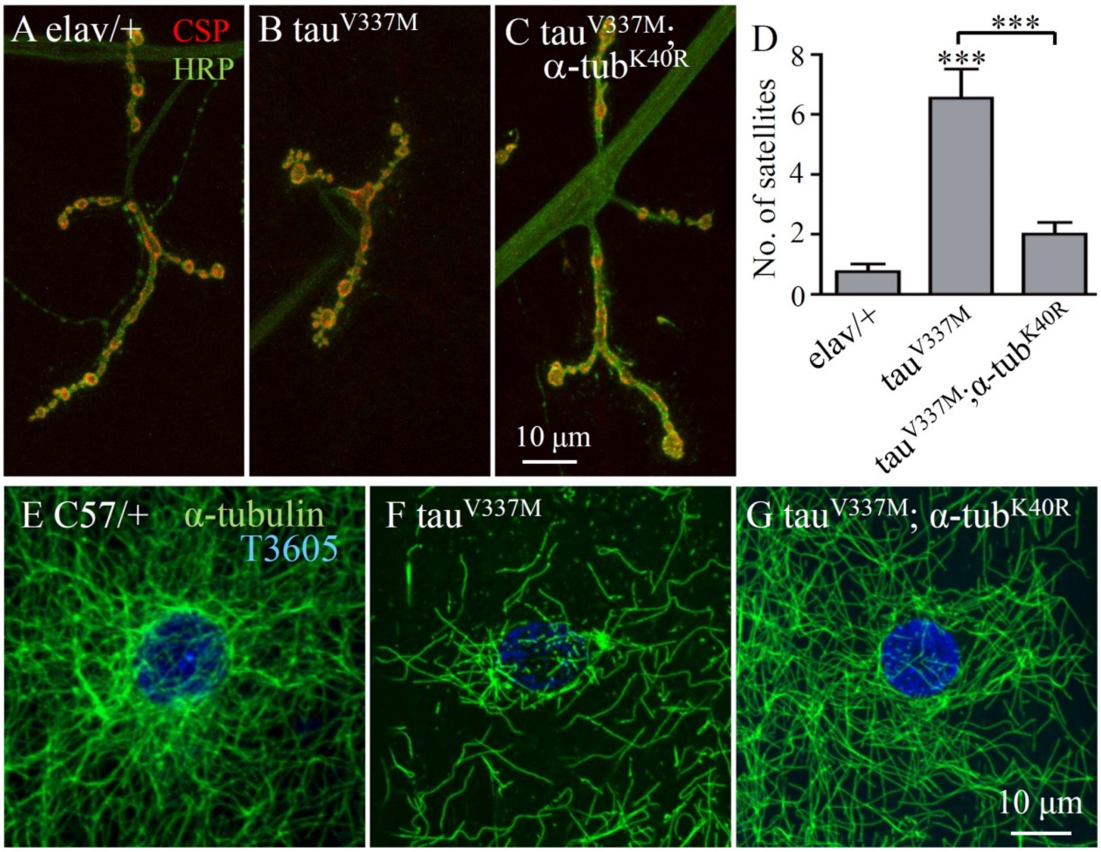

Fig. S3. *α-tub*^{K40R} antagonizes tau toxicity in regulating MT network formation in muscle cells and NMJ growth. (A-C) Representative NMJ4 from wandering 3rd instar larvae double-stained with anti-CSP (red) and anti-HRP (green) to reveal synaptic vesicles and neuronal membrane, respectively. (A) Genetic control *elav-Gal4*/+. (B) Neuronal overexpression of *tau*^{V337M} (*elav-Gal4/tau*^{V337M}). (C) *α-tub*^{K40R} mutation rescued the increased number of satellite boutons caused by neuronal *tau*^{V337M} overexpression(*elav-Gal4/tau*^{V337M}*; α-tub*^{K40R}). (D) Quantification of the number of satellite boutons in different genotypes. *n*=12 NMJs for each genotype. ***$P < 0.001$ (one-way ANOVA). Error bars indicate s.e.m. (E-G) Third instar larval muscles double-stained with anti-tubulin to reveal MT network (green) and T3605 to reveal nucleus (blue). (E) Genetic control *C57-Gal4*/+. (F) Overexpression of tau^{V337M} in muscles (*C57-Gal4/tau*^{V337M}). (G) *α-tub*^{K40R} mutation rescued MT defects caused by *tau*^{V337M} overexpression (*C57-Gal4/tau*^{V337M}*; α-tub*^{K40R}).

Disease Models & Mechanisms • Supplementary information
